# Functional analysis of the bZIP-type transcription factors AtfA and AtfB in *Aspergillus nidulans*

**DOI:** 10.3389/fmicb.2022.1003709

**Published:** 2022-09-20

**Authors:** Beatrix Kocsis, Mi-Kyung Lee, Jae-Hyuk Yu, Tibor Nagy, Lajos Daróczi, Gyula Batta, István Pócsi, Éva Leiter

**Affiliations:** ^1^Department of Molecular Biotechnology and Microbiology, Faculty of Science and Technology, University of Debrecen, Debrecen, Hungary; ^2^ELRN-UD Fungal Stress Biology Research Group, Debrecen, Hungary; ^3^Doctoral School of Pharmaceutical Sciences, University of Debrecen, Debrecen, Hungary; ^4^Biological Resource Center, Korea Research Institute of Bioscience and Biotechnology (KRIBB), Jeongeup-si, South Korea; ^5^Department of Bacteriology, Food Research Institute, University of Wisconsin, Madison, WI, United States; ^6^Department of Systems Biotechnology, Konkuk University, Seoul, South Korea; ^7^Department of Applied Chemistry, Faculty of Science and Technology, University of Debrecen, Debrecen, Hungary; ^8^Department of Solid State Physics, Faculty of Science and Technology, University of Debrecen, Debrecen, Hungary; ^9^Department of Genetics and Applied Microbiology, Faculty of Science and Technology, University of Debrecen, Debrecen, Hungary

**Keywords:** *Aspergillus nidulans*, environmental stress, conidiospore, cleistothecium, sterigmatocystin

## Abstract

Transcription factors (TFs) with the basic leucin zipper domain are key elements of the stress response pathways in filamentous fungi. In this study, we functionally characterized the two bZIP type TFs AtfA and AtfB by deletion (*Δ*) and overexpression (OE) of their encoding genes in all combination: *ΔatfA, ΔatfB, ΔatfAΔatfB, ΔatfAatfB*OE, *ΔatfBatfA*OE, *atfA*OE, *atfB*OE and *atfA*OE*atfB*OE in *Aspergillus nidulans*. Based on our previous studies, *ΔatfA* increased the sensitivity of the fungus to oxidative stress mediated by menadione sodium bisulfite (MSB) and tert-butylhydroperoxide (*t*BOOH), while *ΔatfB* was not sensitive to any oxidative stress generating agents, namely MSB, *t*BOOH and diamide at all. Contrarily, the *ΔatfB* mutant was sensitive to NaCl, but tolerant to sorbitol. Overexpression of *atfB* was able to compensate the MSB sensitivity of the *ΔatfA* mutant. Heavy metal stress elicited by CdCl_2_ reduced diameter of the *atfB*OE and *atfA*OE*atfB*OE mutant colonies to about 50% of control colony, while the cell wall stress generating agent CongoRed increased the tolerance of the *ΔatfA* mutant. When we tested the heat stress sensitivity of the asexual spores (conidiospores) of the mutants, we found that conidiospores of *ΔatfAatfB*OE and *ΔatfBatfA*OE showed nearly 100% tolerance to heat stress. Asexual development was negatively affected by *ΔatfA*, while *atfA*OE and *atfA*OE coupled with *ΔatfB* increased the number of conidiospores of the fungus approximately 150% compared to the control. Overexpression of *atfB* led to a 25% reduction in the number of conidiospores, but increased levels of *abaA* mRNA and size of conidiospores. Sexual fruiting body (cleistothecium) formation was diminished in the *ΔatfA* and the *ΔatfAΔatfB* mutants, while relatively elevated in the *ΔatfB* and the *ΔatfBatfA*OE mutants. Production of the mycotoxin sterigmatocystin (ST) was decreased to undetectable levels in the *ΔatfA* mutant, yet ST production was restored in the *ΔatfAΔatfB* mutant, suggesting that *ΔatfB* can suppress ST production defect caused by *ΔatfA*. Levels of ST were also significantly decreased in the *ΔatfAatfB*OE, *ΔatfBatfA*OE and *atfA*OE*atfB*OE mutants.

## Introduction

Basic-region leucine zipper (bZIP)-type transcription factors contribute to a complex regulatory network to organize differentiation, maintenance of cell types as well as stress responses of eukaryotic organisms. By forming homo-or heterodimers with other bZIP-type transcription factors they coordinate a great variety of cellular processes (Jindrich and Degnan, 2016; Leiter et al., 2021). The *Schizosaccharomyces pombe* Atf1 ortholog bZIP-type transcription factor AtfA regulates several processes including stress tolerance, secondary metabolism and development in vegetative hyphae of numerous filamentous fungi, e.g., *Aspergillus nidulans* (Lara-Rojas et al., 2011), *Neurospora crassa* (Yamashita et al., 2008), *Magnaporthe oryzae* (Guo et al., 2010), *Botrytis cinerea* (Temme et al., 2012), *Fusarium verticillioides* (Szabó et al., 2020). Moreover, AtfA is also involved in the virulence of the human pathogenic *Aspergillus fumigatus* (Silva et al., 2017) and also in the infection of hosts by plant pathogenic fungi, e.g., *Claviceps purpurea* (Nathues et al., 2004), *Magnaporthe oryzae* (Guo et al., 2010), *Botrytis cinerea* (Temme et al., 2012), *Fusarium graminearum* (Nguyen et al., 2013). In *Aspergillus nidulans* the Atf1 ortholog AtfA has been thoroughly characterized (Balázs et al., 2010; Emri et al., 2015; Orosz et al., 2017). AtfA contributes to the vegetative growth and conidiospore formation and also to the tolerance of the fungus to oxidative stress reagents such as menadione sodium bisulfite (MSB) and tert-butylhydroperoxide (*t*BOOH) (Balázs et al., 2010; Emri et al., 2015). Conidia of the *ΔatfA* mutant was also sensitive to osmotic, fungicide and heat stress (Hagiwara et al., 2008, 2009) and their viability were reduced after storage at 4°C (Balázs et al., 2010). Transcriptome based data confirmed that AtfA is important in the regulation of many stress-related and stress-unrelated genes (Emri et al., 2015; Antal et al., 2020) and likely to be involved in the regulation of numerous genes indirectly (Orosz et al., 2017; Antal et al., 2020). Formation of heterodimer of AtfA with other bZIP-type transcription factors, e.g., AtfB was first indicated by Lara-Rojas et al. (2011) in *A. nidulans*. In *Aspergillus oryzae* a transcriptome based study found a set of genes co-regulated by AtfA and AtfB, but AtfA seems to be more important in the regulation of the oxidative stress in this fungus (Sakamoto et al., 2009). In *Aspergillus oryzae* conidia of the *ΔatfA* mutant were more sensitive to oxidative stress than that of the *ΔatfB* mutant (Sakamoto et al., 2009). In *Aspergillus fumigatus* AtfA interacts with AtfB-D transcription factors and coordinate the stress response pathway and virulence of this human pathogenic fungus (Silva et al., 2021). According to the phenotype of the single or double deletion mutants of *atfA* and *atfB* in the presence of different environmental stress agents resulted in either epistatic, additive and suppression interaction of AtfA and AtfB suggests a versatile function of these bZIP transcription factors in *A. fumigatus* (Silva et al., 2021).

In this study, we analyzed the physiological functions of *Aspergillus nidulans atfA* and *atfB* through the construction of gene deletion and overexpression mutants in all combination. Stress sensitivity tests, conidiospore viability, sexual and asexual sporulation as well as sterigmatocystin (ST) production were involved in our phenotypic studies. Based on our observations AtfA seems to be more important in the stress response, conidiospore formation as well as mycotoxin production than AtfB and depending on the tested phenotype *atfB* overexpression can compensate the negative effect of the deletion of *atfA*.

## Materials and methods

### Strains, culture media

*Aspergillus nidulans* strains used in our study is summarized in Supplementary Table S1. All strains were maintained on Barratt’s nitrate minimal medium (NMM) with appropriate nutritional supplements (Barratt et al., 1965), and NMM agar plates were incubated at 37°C for 6 d (Balázs et al., 2010). Conidia harvested from these 6 days old plates were used in all further experiments.

### Construction of gene deletion and overexpression strains

Gene deletion mutants were constructed by the Double-Joint PCR method of Yu et al. (2004) and Leiter et al. (2016) with primers listed in Supplementary Table S2. The amplified deletion cassettes were used to transform rJMP1.59 or TNJ36.1 strain using the Vinoflow FCE lysing enzyme (Szewczyk et al., 2006). Single copy transformants were selected after Southern blot analysis (Király et al., 2020a). To generate overexpression mutants ORFs were amplified with the primers presented in Supplementary Table S2. The amplicons were digested with restriction enzymes as indicated in Supplementary Table S2, and ligated between the *niiA* promoter and the *trpC* terminator in pHS11 (Leiter et al., 2016). Overexpression of the strains was confirmed by rRT-PCR method (Supplementary Figure S1; Király et al., 2020b).

### Stress sensitivity studies

To study the stress sensitivity of the mutant strains, the agar plate assays of Balázs et al. (2010) were adapted. The following stress generating agents were tested: oxidative stress: 2.0 mM diamide (eliciting GSH/GSSG redox imbalance), 0.08 mM menadione sodium bisulfite (MSB, increasing intracellular superoxide level), 0.8 mM *tert*-butyl hydroperoxide (*t*BOOH, triggering lipid peroxidation; Emri et al., 1997; Pócsi et al., 2005); hyperosmotic stress: 1.5 M NaCl and 2.0 M sorbitol; heavy metal stress: 300 μm cadmium chloride (Leiter et al., 2016); cell wall integrity stress: 54 μm CongoRed (an agent known to alter cell wall polymer composition; Leiter et al., 2016). Plates were point-inoculated with 5 μl freshly made conidia suspension (2*10^7^ conidia/ml) and were incubated at 37°C for 5 days (Balázs et al., 2010). In all stress sensitivity studies, the isogenic prototrophized THS30.3 strain was used as the control strain.

### Conidiospore heat stress-sensitivity

To test the heat sensitivity of asexual spores, conidia were harvested from 6 days old colonies and suspended in physiological saline-0.01% Tween 80. Conidia in 10^5^/ml concentration were incubated at 50°C for 10 min and, following that, were diluted and spread on NMM agar plates. The numbers of colonies representing successfully germinated conidia were counted after incubation for 2 days at 37°C. Conidia without any heat treatment were used as reference.

### Sexual and asexual developments

To induce cleistothecium formation, 6 days old conidia were spread in agar at 10^5^ conidia/plate and incubated at 37°C. After 24 h, plates were sealed with Parafilm and samples were taken with a cork borer after 14 days incubation and cleistothecia/cm^2^ were determined under a dissection microscope (Leiter et al., 2016).

The conidiospore forming capabilities of the *A. nidulans* strains were determined as published by Vargas-Pérez et al. (2007). Briefly, conidia (10^5^) of the mutant and control strains were spotted onto NMM agar plates as described above, and were incubated and were allowed to sporulate at 37°C for 5 days. Conidia were washed, counted in a Burker chamber and spore numbers were expressed as number/cm^2^ of colony surface.

### Evaluation of the size of conidiospores

5*10^5^ conidia were spread onto NMM medium, then incubated at 37°C for 5 days. After incubation microscopic images were taken of the conidospores of the mutants and control strain in Burker chamber by ToupView image processing software. Correlated to the known length grid lines of Burker chamber, the size of conidiospores can be calculated. The size of conidiospores was also determined by SEM according to Springer and Yanofksy (1989). Briefly, point-inoculated 5 d old surface cultures were dehydrated stepwise by an ethanol series consisting of 30, 50, 70, 95, and 100% ethanol, 15 min per step. The samples were coated with gold, and observed under a scanning electron microscope (Hitachi S 4300, Schaumburg, United States).

### Sterigmatocystin analysis

Levels of sterigmatocystin (ST) was determined from 5 days old surface cultures according to Yin et al. (2013). A 2 cm^2^ agar plug was removed of each plate culture and extracted with 800 μl by 70% (v/v) acetone. Metabolites were separated in the developing solvent toluene:ethyl acetate:acetic acid (TEA, 8:1:1) on silica coated thin-layer chromatography (TLC) plates and photographs were taken following exposure to UV radiation at 366 nm wavelengths.

The mycelial extracts were also subjected for HPLC analysis. Aliquots of 10 μl were injected into the chromatographic system which consisted of a Waters 2,695 Separations Module equipped with a thermostable autosampler (5°C) and column module (35°C). UV detection was applied by a Waters 2,996 photodiode array detector (254 nm). Separations were performed using an Agilent Zorbax SB-C18 (4.6 mm × 75 mm, 3.5 m) column with 1 ml/min flow rate. Isocratic elution was used where the mobile phase was methanol/acetonitrile/ water 50/15/35 (v/v), respectively (Yin et al., 2013).

### rRT-PCR assays to determine *abaA* gene expression

Total RNA was isolated from surface cultures according to Chomczynski (1993) and rRT-PCR experiments were carried out as described previously (Emri et al., 2015). The applied primer pairs are summerized in Supplementary Table S2. Relative transcript levels were calculated by the ‘delta method’ where ΔC*_P_* = C*_P_* reference gene − C*_P_ abaA* gene and C*_P_* stands for the rRT-PCR cycle numbers corresponding to the crossing points. For statistical analysis, the mean ± SD values were calculated from three independent experiments (Pfaffl, 2001). As reference gene, *actA* (AN6542) was used (Emri et al., 2015).

### Statistical analysis of experimental data

All experiments were performed in three independent sets, and mean ± SD values were calculated and are presented. Statistical significances were calculated using Student’s *t*-test, and *p*-values less than 5% were considered as statistically significant.

## Results

### Stress sensitivity phenotypes of the mutants

**Figure 1:**
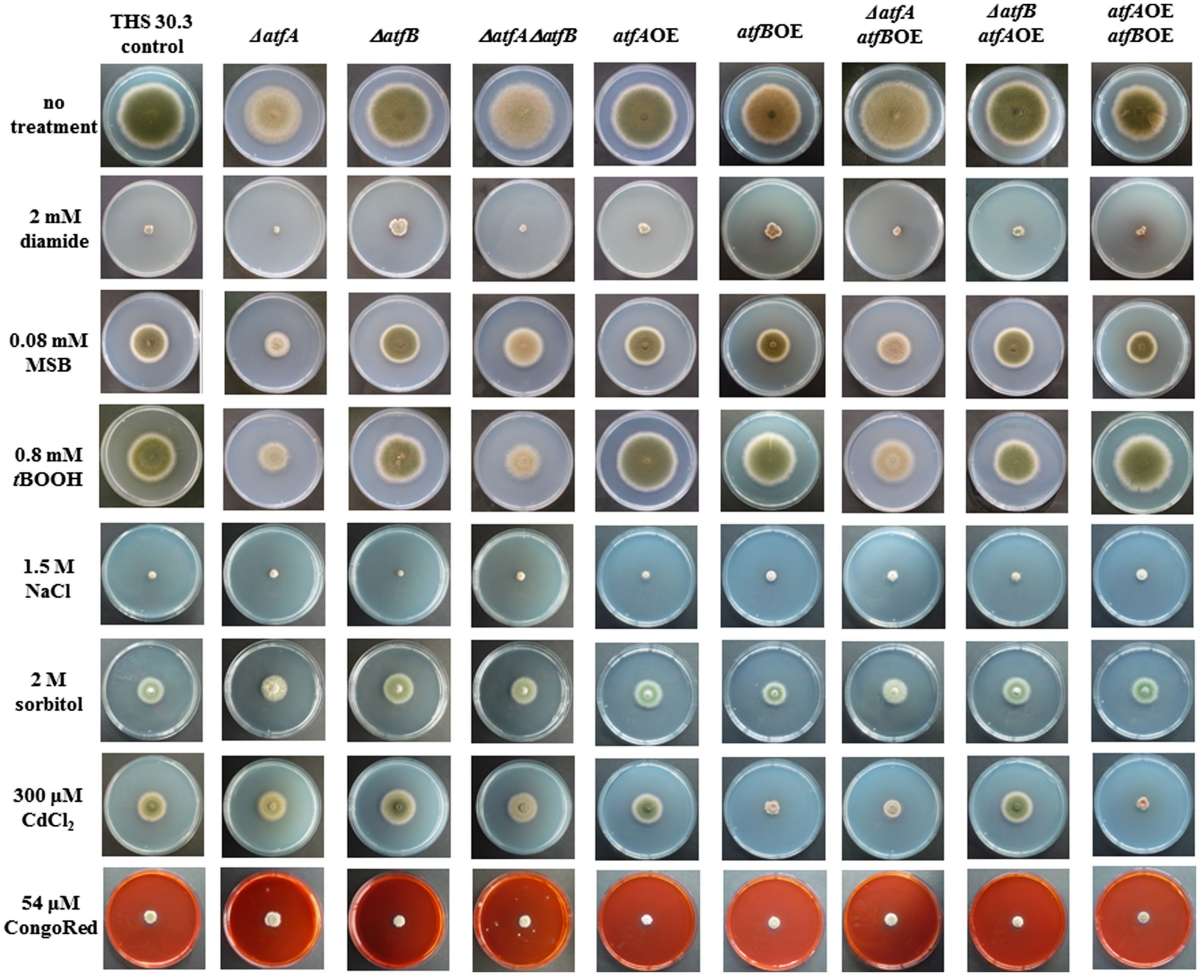
Stress sensitivities of the THS30.3 (control) and the *atfA* and *atfB* gene deletion (*Δ*) and overexpression (OE) mutant strains exposed to various types of stress reagents. Stress sensitivities observed in surface cultures on NMM agar plates are shown. NMM agar plates were incubated at 37°C for 5 days.

*ΔatfA, atfB*OE and *atfA*OE*atfB*OE strains showed reduced growth compared to the control strain on minimal medium at 37°C without any stress treatment. Increased sensitivity to oxidative stress inducing agent diamide was observed in the *ΔatfAatfB*OE and *ΔatfAΔatfB* as well as in the *atfA*OE*atfB*OE strains. Interestingly both the deletion and overexpression of *atfB* increased the diamide tolerance of the fungus. MSB sensitivity was detected only in the *ΔatfA* strain and *atfBOE* was able to compensate this stress sensitivity in the *ΔatfAatfB*OE mutant with approximately doubled colony growth compared to the control. Overexpression of *atfA*, *atfB* alone and together increased the *t*BOOH tolerance of the fungus with approximately 20%, while *ΔatfA* reduced the growth of *A. nidulans* to 50% in the presence of *t*BOOH. In the *ΔatfB* mutant compared to the control there was no difference in the *t*BOOH sensitivity, therefore the *t*BOOH sensitivity of the double deletion mutant is as a result of the deletion of *atfA* (Figure 1). To study the osmotic stress sensitivity we tested our mutants in the presence of 1.5 M NaCl and 2 M sorbitol. 1.5 M NaCl significantly reduced the growth of the *ΔatfB* mutant compared to the control, while the double overexpression mutant showed increased the tolerance to NaCl. Surprisingly, the *ΔatfB* mutant was the most tolerant while the *atfB*OE was the most sensitive to 2 M sorbitol compared to the other strains (Figure 1). The heavy metal stress sensitivity was tested in the presence of 300 μm CdCl_2_. The *ΔatfAatfB*OE mutant showed slightly reduced growth, while the diameter of the colony growth of *atfB*OE and *atfA*OE*atfB*OE mutant was half of that of the control strain exposed to CdCl_2_. Contrarily, the *ΔatfB* mutant was moderately tolerant to CdCl_2_ (Figure 1). Only the *ΔatfA* mutant was affected to the exposure to 54 μm CongoRed and showed moderate tolerance (Figure 1).

### Heat stress-sensitivity of the conidiospores

**Figure 2:**
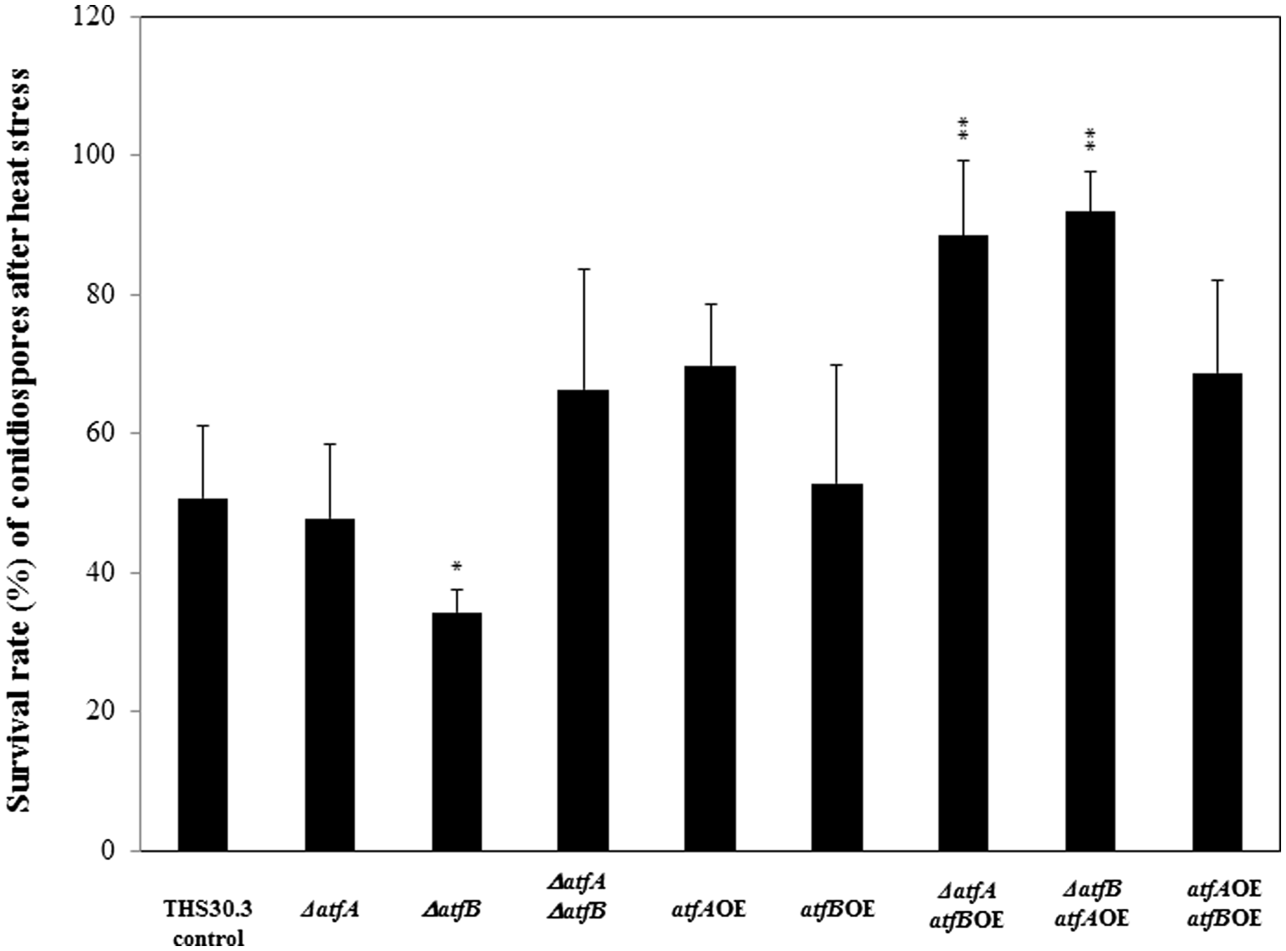
Heat stress sensitivity of conidiospores of the control and mutant strains. Conidia without heat treatment (50°C for 10 min) were used as control. Data are presented as mean ± SD values calculated from three independent experiments. Significant differences between control and mutant cultures (*p < 5%, **p < 1%) are indicated.

We tested the viability of the conidiospores under heat stress. Incubation of conidiospores of the *ΔatfAatfB*OE and *ΔatfBatfA*OE mutant at 50°C for 10 min resulted in increased viability - survival rates of the conidiospores were nearly 100% -, meanwhile the asexual spores of the *ΔatfB* showed reduced viability after heat stress compared to the control strain (Figure 2). We did not find any differences in the heat stress sensitivity of the other mutants and the control strain.

### Sexual and asexual developments

**Figure 3:**
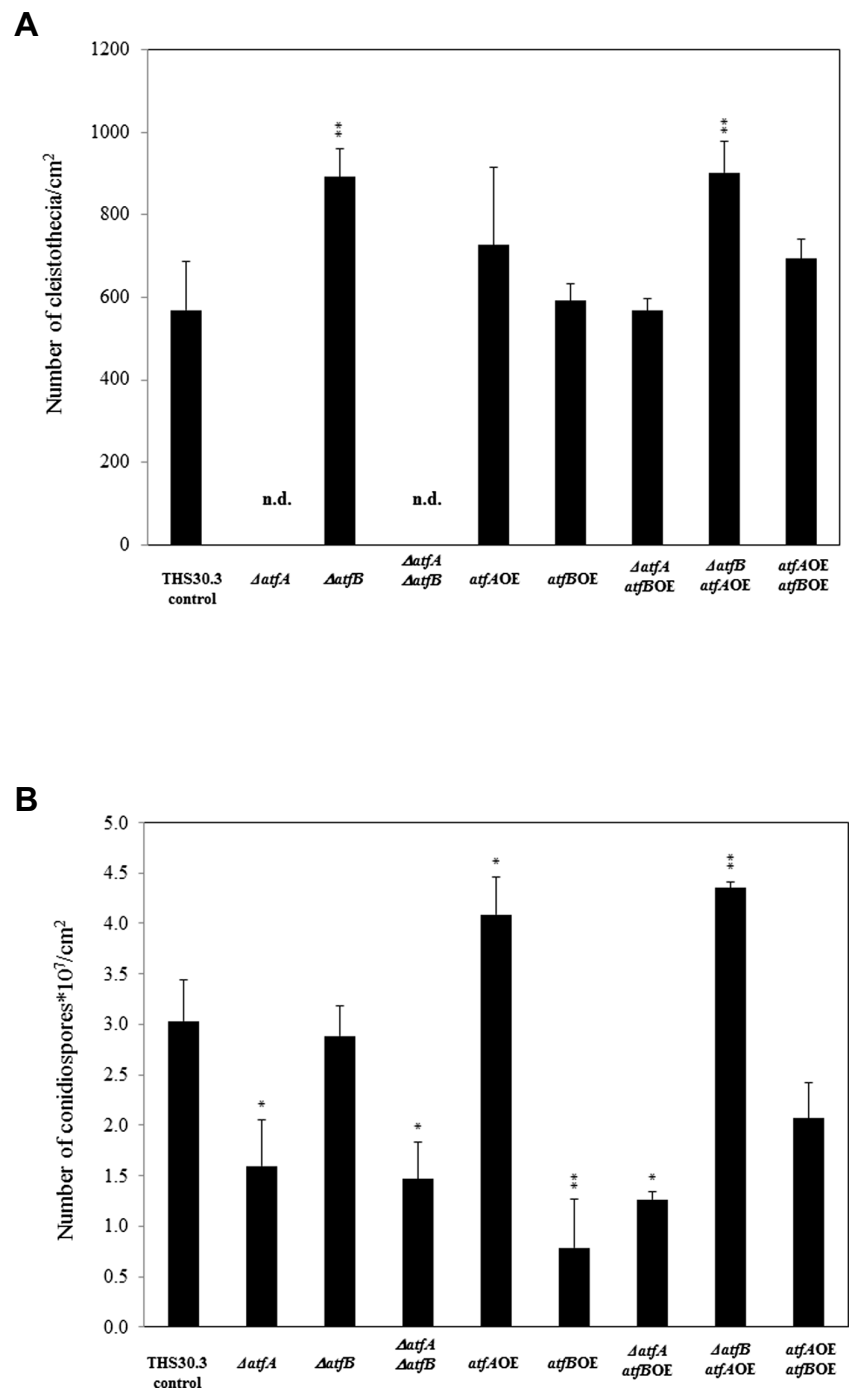
Cleistothecia and conidiospore productions of the control and mutant strains. **(A)** Cleistothecia productions observed after 14 days incubations. **(B)** Conidiospore productions. Data are presented as mean ± SD values calculated from three independent experiments. Significant differences between control and mutant cultures (*p < 5%, **p < 1%) are presented. n.d., not detected.

We also quantified cleistothecia formation and conidiospore production in all mutants. Deletion of *atfA* and *atfA*, *atfB* together inhibited the cleistothecium formation, while in *ΔatfB* and *ΔatfBatfA*OE mutants approximately one and the half times higher fruiting body formation was observed compared to the control (Figure 3A).

Deletion of *atfA* significantly decreased the number of conidiospores both in the *ΔatfA* as well as in the *ΔatfAΔatfB* mutants. In the *atfB*OE and *ΔatfAatfB*OE mutants also reduced conidiospore formation was observed. The overexpression of *atfA* alone and in the *ΔatfB* background increased the number of asexual spores of the fungus with nearly one and the half times (Figure 3B).

### Evaluation of the size of conidiospores and *abaA* expression

**Figure 4:**
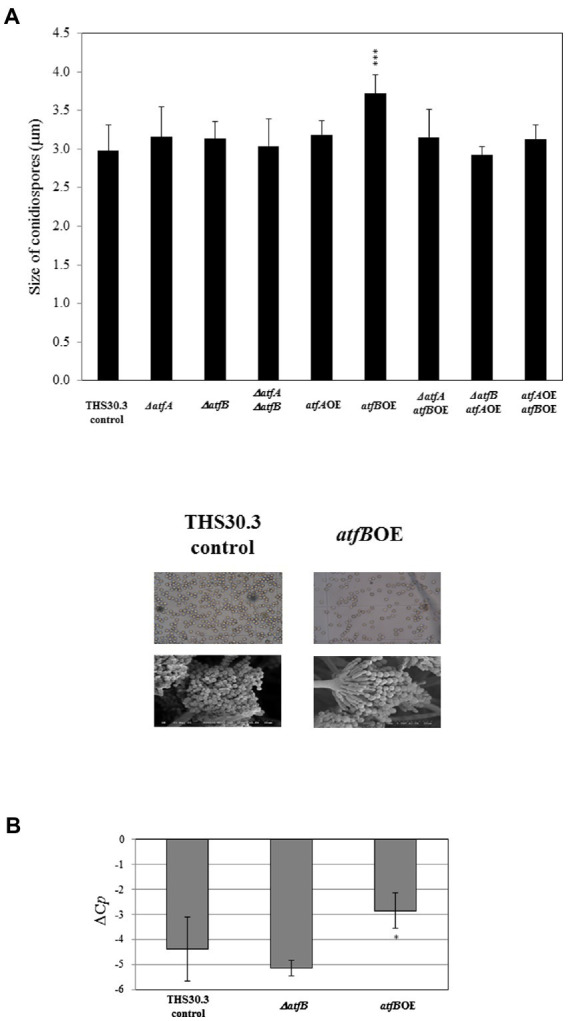
Determination of the size of conidiospores of the control and mutant strains **(A)**. Evaluation of *abaA* gene expression in the control, *ΔatfB* and *atfB*OE mutants **(B)**. Data are presented as mean ± SD values calculated from three independent experiments. Significant differences between control and mutant cultures (*p < 5% and ***p < 0.1%) are presented.

We determined the size of conidiospores by light microscopy and SEM. We found that *atfBOE* mutant produced significantly larger conidiospores compared to the control strain (Figure 4A). We did not find any differences in the size of asexual spores in the rest of the mutants compared to the control strain. We also evaluated the *abaA* (element of the central regulatory pathway of conidiogenesis) gene expression of the surface cultures of the mutants. *abaA* was upregulated in the *atfB*OE mutant compared to the control, but there was no significant differences in the *abaA* expression between the control and *ΔatfB* gene deletion mutant (Figure 4B).

### Sterigmatocystin determination

**Figure 5:**
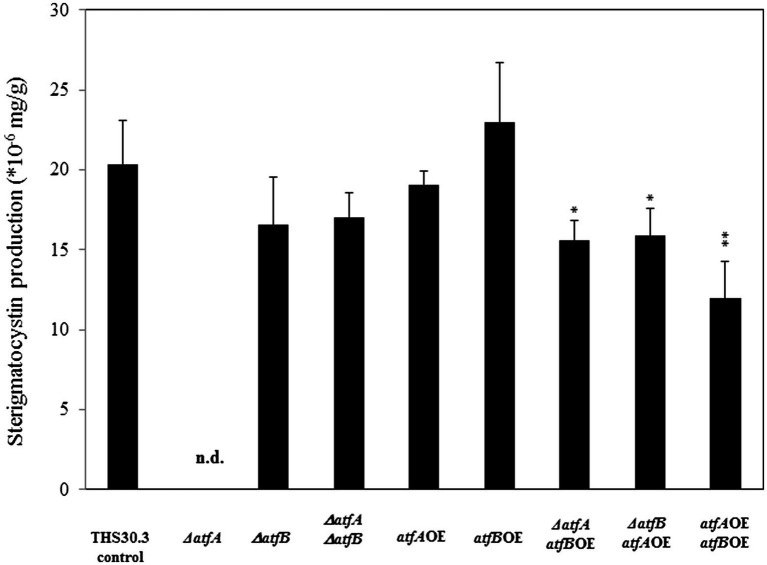
Sterigmatocystin production of the control and mutant strains. Data are presented as mean ± SD values calculated from three independent experiments. Significant differences between control and mutant cultures (*p < 5%, **p < 1%) are indicated. n.d., not detected.

Sterigmatocystin production was determined from 5 days old surface cultures. Deletion of *atfA* resulted in a remarkable reduction of sterigmatocystin production (Figure 5). Interestingly deletion of both *atfA* and *atfB* together did not affect the sterigmatocystin biosynthesis compared to the control. We found decreased sterigmatocystin level in the *ΔatfAatfB*OE and *ΔatfBatfA*OE mutants, and also in the *atfA*OE*atfB*OE mutant where sterigmatocystin concentration was approximately half of that of the control strain.

## Discussion

It is well known that bZIP type transcription factors are important elements of the stress signaling pathway, reproduction and secondary metabolite production in filamentous fungi (Bayram et al., 2008; Jindrich and Degnan, 2016; Leiter et al., 2021). In this study we constructed a series of gene deletion and overexpression mutants of *atfA* and *atfB* either alone or in combination to understand how these bZIP-type transcription factors regulates the stress tolerance, sexual and asexual reproduction and sterigmatocystin production in *Aspergillus nidulans*.

We managed to confirm previous observations that AtfA is involved in the oxidative stress defense system of *Aspergillus nidulans* (Figure 1; Hagiwara et al., 2008, 2009; Balázs et al., 2010; Emri et al., 2015). Deletion of *atfA* resulted in reduced growth in the presence of oxidative stress generating agents, e.g., diamide, *t*BOOH and menadione (Figure 1). In this work further functions of AtfA were unfolded. Deletion of *atfA* inhibited the cleistothecia production completely (Figure 3A) suggesting the outstanding role of AtfA in sexual reproduction of *Aspergillus nidulans*. It is well known that bZIP transcription factors play crucial role in the sexual development of filamentous fungi (Bayram et al., 2008; Yin et al., 2013). For example, Yin et al. (2013) confirmed that overexpression of *rsmA* (restorer of secondary metabolism A), a Yap-like bZIP showed near loss of ascospore production. In fungi sexual reproduction is coupled with secondary metabolism by the Velvet Complex (Bayram et al., 2008). For example, overexpression of *rsmA* increased the ST production with 100 fold in *A. nidulans* (Yin et al., 2013). Relation of secondary metabolism and sexual development was also described in the *napA* overexpression mutant (Yin et al., 2013). NapA similarly to AtfA and AtfB belongs to the Yap-family proteins (Yin et al., 2013). This correlation was also verified in our study since the *ΔatfA* mutant showed failure in fruting body formation and also loss of sterigmatocystion production (Figure 5). Similar phenotype was also observed in *F. verticillioides*, where the deletion of *FvatfA* inhibited fumonisin production (Szabó et al., 2020).

Based on our results AtfB seems to be more important in the heat stress sensitivity, CdCl_2_ sensitivity (Figure 1) and number (Figure 3B) and size of conidiospores than AtfA (Figure 4). Overexpression of *atfB* decreased the tolerance of the fungus to CdCl_2_ (Figure 1). Genome wide expression study by Emri et al. (2021) in *A. nidulans* confirmed that exposure to CdCl_2_ downregulates *atfB* gene expression in the control strain, while no alteration of the *atfB* expression was observed in the *ΔatfA* mutant (transcriptome data accession number: GSE166128). Overexpression of *atfB* decreased the number and increased the size of asexual spores (Figures 3B, 4) and also increased the *abaA* gene expression (Adams et al., 1998). In *Beauveria bassiana*, a filamentous entomopathogen deletion of *wetA* resulted in 90% repression of *abaA* gene expression and concomitantly smaller size of conidia (Li et al., 2015). In *Fusarium graminearum* overexpression of *abaA* caused in pleiotropic defects such as impaired sexual and asexual development, delayed conidium germination, and decreased trichothecene production (Son et al., 2013). In *Aspergillus fumigatus* overexpression of *AfuabaA* resulted in autolysis and cell death (Tao and Yu, 2011). Similarly, in *Aspergillus oryzae* AtfB is also important in the production of conidia (Sakamoto et al., 2008). Under osmotic stress conditions *ΔatfB* produced less conidia in *A. oryzae* suggesting the role of *atfB* in the development of conidiospores as well (Sakamoto et al., 2008, 2009). In our study, *ΔatfB* was sensitive to heat stress similarly to the *ΔatfB* in *A. oryzae* (Sakamoto et al., 2008; Figure 2).

Analysis of the phenotype of the mutants where both *atfA* and *atfB* were manipulated genetically indicates that some of the physiological functions of *Aspergillus nidulans* are coordinated by both of the bZIPs. For example, we observed the highest heat stress tolerance in the *ΔatfAatfB*OE and *ΔatfBatfA*OE strains compared to those of the rest of the mutants and the control strain (Figure 2). No fruiting body formation was observed in the *ΔatfAΔatfB* double deletion mutant, but more cleistothecia were produced in the *ΔatfBatfA*OE strain compared to the control, but the number of cleistothecia of *ΔatfBatfA*OE and *ΔatfB* was similar (Figure 3A). Surprisingly, when *atfB* was also deleted in the *ΔatfA* mutant sterigmatocystin production was similar to that of the control strain and in the *atfA*OE*atfB*OE mutant we observed less toxin production than in the *atfA*OE and *atfB*OE mutants (Figure 5) suggesting that toxin production is likely under the control of both bZIPs in *A. nidulans*.

bZIP transcription factors can form homodimers with themselves and heterodimers with other bZIPs and may also interact physically with stress signaling proteins as well (Lara-Rojas et al., 2011; Silva et al., 2021). For example, in *A. fumigatus* AtfA physically interacts with other three bZIP transcription factors, namely AtfB, AtfC and AtfD as well as with the MAPK SakA to coordinate stress response (Silva et al., 2021). In contrast to our observation the double deletion mutant *ΔatfAΔatfB* was as sensitive as MSB as the corresponding single mutants in *A. fumigatus*. Both *ΔatfA* and *ΔatfB* was as sensitive as *ΔatfAΔatfB* to the cell wall stress generating agents calcofluor white (CFW) and CongoRed (Silva et al., 2021).

Based on our results AtfA and AtfB may interact with each other to coordinate expressions of genes involved in the stress tolerance, sexual and asexual development as well as secondary metabolite production in *A. nidulans*. To confirm this hypothesis further studies, e.g., BiFC experiments are in progress in our laboratory.

## Data availability statement

The raw data supporting the conclusions of this article will be made available by the authors, without undue reservation.

## Author contributions

J-HY, IP, and ÉL: conceptualization and writing. BK, M-KL, TN, LD, and GB: methodology. All authors discussed the review and contributed to the final manuscript.

## Funding

The research was supported by the European Union and the European Social Fund through the project EFOP-3.6.1-16-2016-00022, Thematic Excellence Programme (TKP2021-EGA-20; Biotechnology) of the Ministry for Innovation and Technology in Hungary, and National Research, Development and Innovation Office with the grants NKFIH K119494 and NN125671. The work at UW-Madison was supported by the UW Food Research Institute.

## Conflict of interest

The authors declare that the research was conducted in the absence of any commercial or financial relationships that could be construed as a potential conflict of interest.

## Publisher’s note

All claims expressed in this article are solely those of the authors and do not necessarily represent those of their affiliated organizations, or those of the publisher, the editors and the reviewers. Any product that may be evaluated in this article, or claim that may be made by its manufacturer, is not guaranteed or endorsed by the publisher.
