# Supplementary material for: Functional analysis of the bZIP-type transcription factors AtfA and AtfB in Aspergillus nidulans
Source: Front Microbiol. 2022 Sep 20;13:1003709. doi: 10.3389/fmicb.2022.1003709 (PMC9530789; doi:10.3389/fmicb.2022.1003709)
Supplement: Supplementary file 1 [file Table_1.DOC]

**Table S1.** *Aspergillus* strains used in this study.

| **Strain** | **Genotype** | **Origin/Reference** |
| --- | --- | --- |
| *A. nidulans* |  |  |
| RJMP 1.59 | *pyrG89; pyroA4; veA^+^* | Shaaban et al. |
| TNJ36.1 | *pyrG89; pyroA4; pyrG^+^; veA^+^* | Kong et al. |
| THS30.3 (control strain) | *pyrG89*; *pyroA^+^*; *veA^+^*; *AfupyrG^+^* | Emri et al. |
| *ΔatfA* | *pyrG89*; *ΔatfA*::*AfupyrG*^+^; *pyroA^+^*; *veA*^+^) | This study |
| *ΔatfB* | *pyrG89*; *ΔatfB*::*AfupyrG*^+^; *pyroA^+^*; *veA*^+^ | This study |
| *ΔatfAΔatfB* | *pyrG89*; *pyroA4*; *ΔatfB*::*AfupyrG*^+^; *ΔatfA::pyroA^+^*; *veA*^+^ | This study |
| *atfA*OE | *pyroA4; niiA(p)::atfA::pyroA3/4* | This study |
| *atfB*OE | *pyroA4; niiA(p)::atfB::pyroA3/4* | This study |
| *atfA*OE*atfB*OE | *pyroA4; niiA(p)::atfA::pyroA3/4; pyrG89; niiA(p)::atfB::AfupyrG* | This study |
| *atfA*OE*ΔatfB* | *pyroA4; niiA(p)::atfA::pyroA3/4; pyrG89*; *ΔatfB*::*AfupyrG*^+^; *pyroA^+^*; *veA*^+^ | This study |
| *atfB*OE*ΔatfA* | *pyroA4; niiA(p)::atfB::pyroA3/4; pyrG89*; *ΔatfA*::*AfupyrG*^+^; *pyroA^+^*; *veA*^+^ | This study |
| *A. fumigatus* |  |  |
| Af293 | Wild type | Brookman and Denning, 2000 |

**Supplementary References**

Shaaban, M. I., Bok, J. W., Lauer, C. & Keller, N. P. Suppressor mutagenesis identifies a velvet complex remediator of *Aspergillus nidulans* secondary metabolism. *Eukaryot Cell* **9,** 1816-1824 (2010).

Kong, Q. et al Gβ-like CpcB plays a crucial role for growth and development of *Aspergillus nidulans* and *Aspergillus fumigatus*. *PLoS One* 8, e70355 (2013).

Emri, T. et al. Core oxidative stress response in *Aspergillus nidulans*. *BMC Genomics* **16,** 478 (2015).

Brookman, J. L. and Denning, D. W. Molecular genetics in *Aspergillus fumigatus*. *Curr Opin Microbiol* **3,** 468-474 (2000).
