# Supplementary material for: Functional analysis of the bZIP-type transcription factors AtfA and AtfB in Aspergillus nidulans
Source: Front Microbiol. 2022 Sep 20;13:1003709. doi: 10.3389/fmicb.2022.1003709 (PMC9530789; doi:10.3389/fmicb.2022.1003709)
Supplement: Supplementary file 2 [file Table_2.DOC]

**Table S2.** Oligonucleotides used in this study.

| Name | Sequence (5’→3’) | Purpose |
| --- | --- | --- |
| OMK830 | TTCGAGTGGCAATCTGATAAC | 5’-flanking region of *atfA* |
| OMK831 | GCTTTGGCCTGTATCATGACTTCAAGTTCCCCCCGCGATCCGGTC | 5’ *atfA* with *AfupyrG* tail |
| OMK832 | ATCGACCGAACCTAGGTAGGGTAAGGAATAATATCACTTGACTAAC | 3’ *atfA* with *AfupyrG* tail |
| OMK833 | AATATACAGTCCAAAAAGGC | 3’-flanking region of *atfA* |
| OMK834 | TCCATAAATAAACACTCCGACCA | 5’ nested of *atfA* |
| OMK835 | AACCGGGAGAGCCACAACAT | 3’ nested of *atfA* |
| OMK589 | GCTGAAGTCATGATACAGGCCAAA | 5' *AfupyrG* marker |
| OMK590 | ATCGTCGGGAGGTATTGTCGTCAC | 3' *AfupyrG* marker |
| OMK839 | AGTTCCCCCCGCGATCCGGTCACTTCTGCAGTCGGAATTGGCCTG | 5’ *atfA* with *pyroA* tail |
| OMK840 | TGGTGAGAACACATGCACAACTTGAGGAATAATATCACTTGACTAAC | 3’ *atfA* with *pyroA* tail |
| ONK395 | ATCTCATGGGTGCTGTGCGAAAGG | 5' *AnipyroA* marker |
| ONK396 | TTGCATCGCATAGCATTGCATTGC | 3' *AnipyroA* marker |
| OHS966 | AAGCTCCTCTGTCCATCACGATG | 5’-flanking region of *atfB* |
| OHS968 | GCTTTGGCCTGTATCATGACTTCACTGTTGGTTACCAATACTGTTCCT | 5’ *atfB* with *AfupyrG* tail |
| OHS969 | ATCGACCGAACCTAGGTAGGGTACGTTTATCCATTCTCTCCGGCTTC | 3’ *atfB* with *AfupyrG* tail |
| OHS967 | GCGTCTTGTCGTCTGCTCAGATC | 3’-flanking region of *atfB* |
| OHS970 | CCAGGATGTAGTGAGTTGCAGGA | 5’ nested of *atfB* |
| OHS971 | CGAAACGCACCCGCACATAAC | 3’ nested of *atfB* |
| OBK1 | aagaattcATGTCTGCCGCCGTGGCTTCG | 5’ *atfA* with *EcoRI* |
| OBK2 | ggcatatgTCAAGTGTATGGAGGATTCGGG | 3’ *atfA* with *NdeI* |
| OBK3 | aaggatccATGACCAGCCAAACAACTTTC | 5’ *atfB* with *BamHI* |
| OBK4 | ggaagcttCTAAGAAATGCTAATTTGCAGG | 3’ *atfB* with *HindIII* |
| AN6542F | GAAGTCCTACGAACTGCCTGATG | forward primer of the reference gene (*actA*) used for rRT-PCR |
| AN6542R | AAGAACGCTGGGCTGGAA | reverse primer of the reference gene (*actA*) used for rRT-PCR |
| AN0422F | GAAGCGAAGGACCAGGAAAC | forward primer of the *abaA* gene used for rRT-PCR |
| AN0422R | GCGTGAGTGCCGTAGTGAC | reverse primer of the *abaA* gene used for rRT-PCR |
