# Supplementary figures and images for: Functional analysis of the bZIP-type transcription factors AtfA and AtfB in Aspergillus nidulans
Source: Front Microbiol. 2022 Sep 20;13:1003709. doi: 10.3389/fmicb.2022.1003709 (PMC9530789; doi:10.3389/fmicb.2022.1003709)

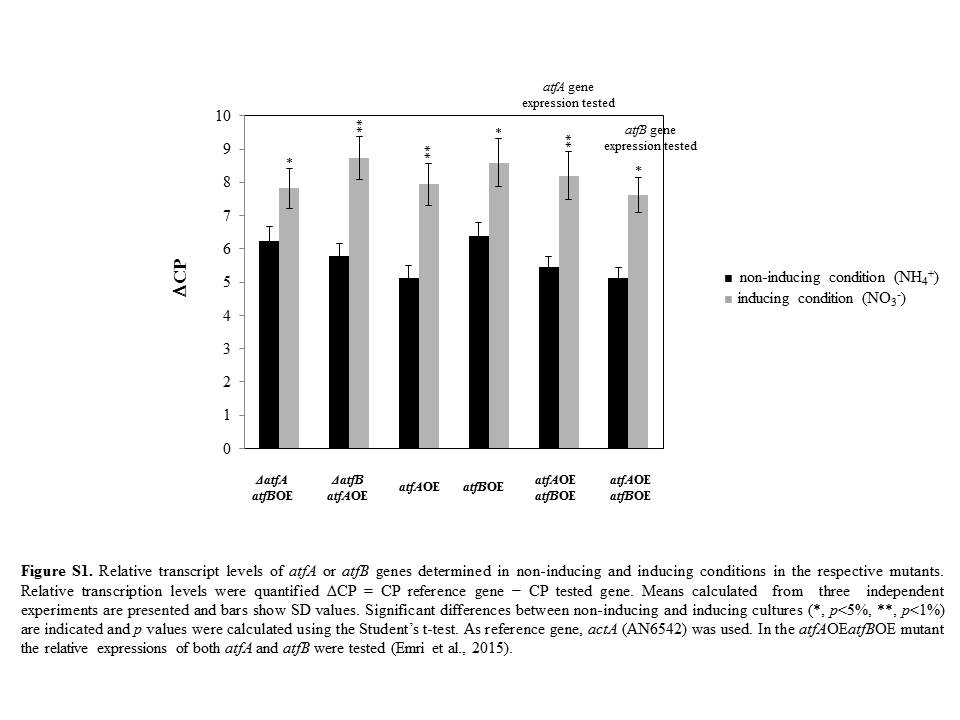

Supplement: Supplementary file 3 [file Image_1.JPEG]
